# Radiation Oncology Resident and Program Director Perceptions of the Job Market and Impact on Well-being

**DOI:** 10.7759/cureus.7187

**Published:** 2020-03-05

**Authors:** Shilo Lefresne, Joshua Giambattista, Paris-Ann Ingledew, Hannah Carolan, Robert A Olson, Shaun Loewen

**Affiliations:** 1 Radiation Oncology, British Columbia Cancer Agency, Vancouver, CAN; 2 Radiation Oncology, Allan Blair Cancer Centre, Regina, CAN; 3 Radiation Oncology, British Columbia Cancer Agency, Prince George, CAN; 4 Radiation Oncology, University of Calgary, Calgary, CAN

**Keywords:** radiation oncology, job market, resident training, well-being, quality of life

## Abstract

Background

Radiation oncology graduates occasionally experience difficulties obtaining employment. The purpose of this study was to explore the perceptions of radiation oncology residents (RORs) and program directors (PDs) about the job market and the potential impact on their well-being.

Methods

RORs and PDs from 13 Canadian training programs were invited to participate. Semi-structured interviews were conducted from March 2014 to January 2015. Knowledge/perception of the job market, impact on personal/professional life, as well as opinions regarding possible contributing factors/solutions to the job market were assessed. A conventional content analysis of each transcript was performed with the clustering of conceptually similar expressions into themes. Demographic information was summarized with descriptive statistics.

Results

Twenty RORs and four PDs participated. All the participants described delayed retirement and over-training as contributors to the job shortage. The majority of trainees interviewed were concerned about the job market (60%) and reported that it impacted their personal (60%) and professional (55%) relationships. PDs described the job market as negatively impacting their job satisfaction. Resident morale was ranked as poor by both groups.

Conclusions

Job market shortages can negatively impact the personal and professional well-being of trainees and PDs. Attention to manpower planning is important to maintaining a high-quality workforce. The cyclical undersupply and oversupply of residents occur in several countries, which makes our findings potentially relevant to residency training programs internationally.

## Introduction

The Canadian radiation oncology job market has been susceptible to under- and oversupply since the 1980s. In 1981, a shortfall of 73 radiation oncologists led to an increase in recruitment and residency training [1]. By the 1990s, patient wait-times increased to “crisis proportions” [2]. In 1997, however, a lack of coordinated planning led to another “looming crisis” in which a 40% unemployment rate was predicted [3]. In 1997, when the unemployment rates were high, over half of the residency positions went unmatched, and some radiation oncology residents (RORs) transferred to different specialties [3,4]. In response, the Canadian Association of Radiation Oncology (CARO) Human Resources and Standards Committee was formed with the task of matching training positions with workforce demands [4]. Despite these efforts, two Canadian surveys in 2013 and 2014 identified that over 50% of radiation oncology graduates were unemployed. They also reported that most graduates were obtaining locum or staff positions approximately two years after graduation, and 26% of these were employed outside Canada [5,6].

The impact of the job market on Canadian RORs has been indirectly assessed in two prior trainee surveys. In 2003, more than 90% of residents were satisfied with their career choice, but almost 50% felt that their program should address job availability [7]. Six years later, as prospects worsened, career satisfaction decreased to 80%; the most commonly cited weakness of training programs was a lack of preparation for entering the job market, and the top source of stress was the shortage of job prospects [8].

Unemployment and underemployment of recent graduates are not unique to radiation oncology. The Royal College of Physicians and Surgeons of Canada (RCPSC) employment study in 2013 identified that resource-intensive specialties were most significantly impacted by job shortages with respondents in cardiac surgery (100%), nuclear medicine (57%), and ophthalmology (43%) self-reporting to be facing the highest rates of difficulty finding full-time employment [5]. While multiple organizations have characterized the job shortages impacting their respective specialties, little has been done to assess the potential impact of these shortages on resident professional and personal well-being. Insight into this area is important for both undergraduate and postgraduate medical educators as they aim to provide mentorship for their trainees during a time when requests for career counselling are becoming more pronounced among Canadian residents [9]. The purpose of this study was to answer the following questions: (1) Do RORs and program directors (PDs) believe there is a job market problem, and if so, what are the possible contributors to and perceived solutions for the problem? (2) Does the current job market impact the personal/professional relationships and well-being of residents and PDs?

## Materials and methods

RORs and PDs from all 13 Canadian training programs were invited to participate in this study via e-mail in February 2014. This project was approved by the University of British Columbia Research Ethics Board. Written informed consent was individually obtained from all participants included in the study.

**Figure 1:**
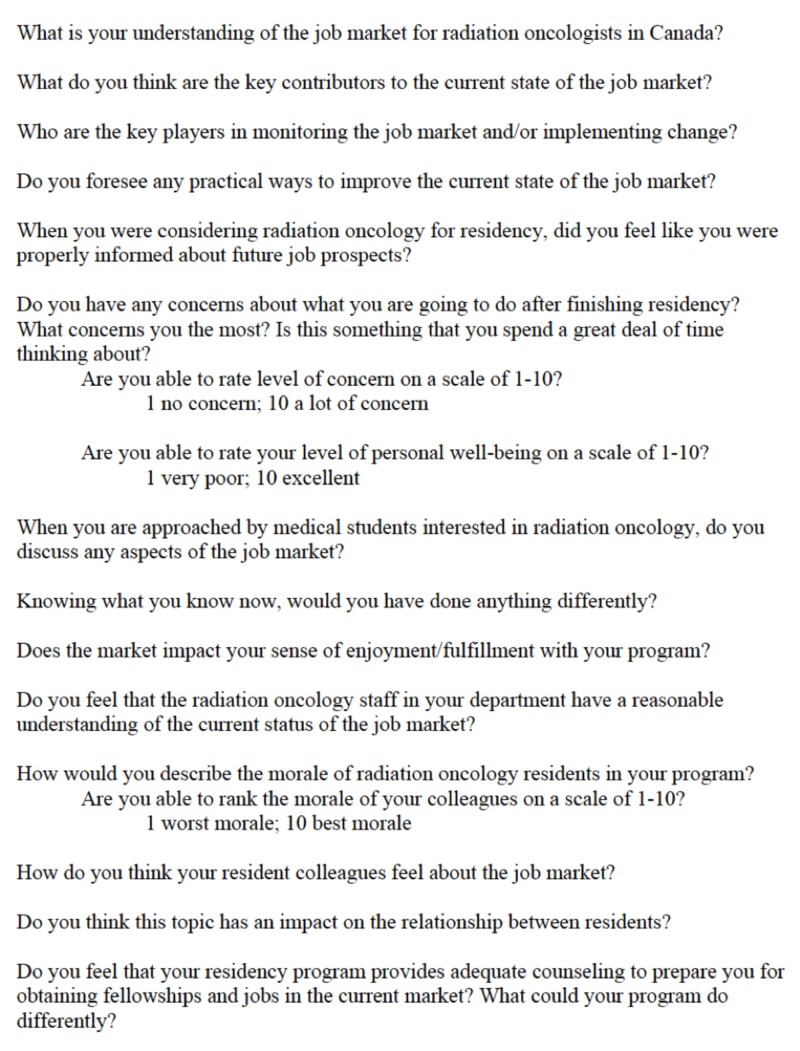
Interview questions for radiation oncology residents Note: demographic questions are not included. Questions for program directors are of similar format

A literature review and information gathered from manpower planning presentations and resident career counselling sessions at the CARO annual general meetings between 2011 and 2013 were used to develop the interview questions. The literature review was performed on MEDLINE using the heading “radiation oncology” and subheading “manpower.” “Radiation oncology” was also linked with keywords “resident” and “quality of life.” Questions were developed by a single investigator with the goal of collecting basic demographic information, assessing ROR knowledge/perception of the job market, impact on personal/professional life and opinions regarding possible contributing factors to and solutions for the current job market situation using open-ended questions (Figure 1). Similar questions were designed for PDs to assess their perceptions about the job market and impact on their personal job satisfaction. In addition, PDs’ observations of how the job market may impact the resident quality of life were assessed in an effort to facilitate the description of the themes identified in ROR interviews through triangulation. Questions for RORs and PDs were disseminated to three other investigators for review and consensus. 

Participants were interviewed from March 2014 to January 2015 either in person or over Skype, Facetime, or telephone by three different interviewers. Interviews were semi-structured with probing for clarification by the interviewer as required and typically lasted 30-40 minutes. The dialogue was audio-recorded then transcribed. Due to the limited literature available on our area of interest, a conventional content analysis of the transcripts was performed in which significant expressions were identified and clustered as they emerged from the data rather than being defined apriori [10]. Each transcript was first reviewed in its entirety. Specific words and statements that described key concepts were highlighted by at least two investigators. Conceptually similar expressions between transcripts were then clustered into themes. Discrepancies in identified themes were discussed among three investigators and resolved by consensus. Data analysis was conducted using NVivo qualitative analysis software, v11.0 (QSR International, Doncaster, Australia). Demographic information was summarized with descriptive statistics.

## Results

Residents

Demographics

Twenty of the 113 (18%) eligible residents from six of the 13 (46%) training programs across the country participated. Table 1 describes participant demographics. There were slightly more junior than senior residents (55% vs 45%) and more participants from Eastern than Western Canada (55% vs 45%); 80% intended to pursue a fellowship after graduation, and 45% were willing to consider employment outside of Canada.

**Table 1:** Basic demographics of 20 radiation oncology residents interviewed

Median age in years (range)	29 (26-34)
Males	12 (60)
Postgraduate year of training (PGY), n (%)	
PGY 1	4 (20)
PGY 2	5 (25)
PGY 3	2 (10)
PGY 4	3 (15)
PGY 5	6 (30)
Province of training, n (%)	
British Columbia	6 (30)
Alberta	2 (10)
Manitoba	1 (5)
Ontario	11 (55)
Residents with a partner, n (%)	18 (90)
Residents with children, n (%)	2 (10)

Perceptions of the Job Market

**Figure 2:**
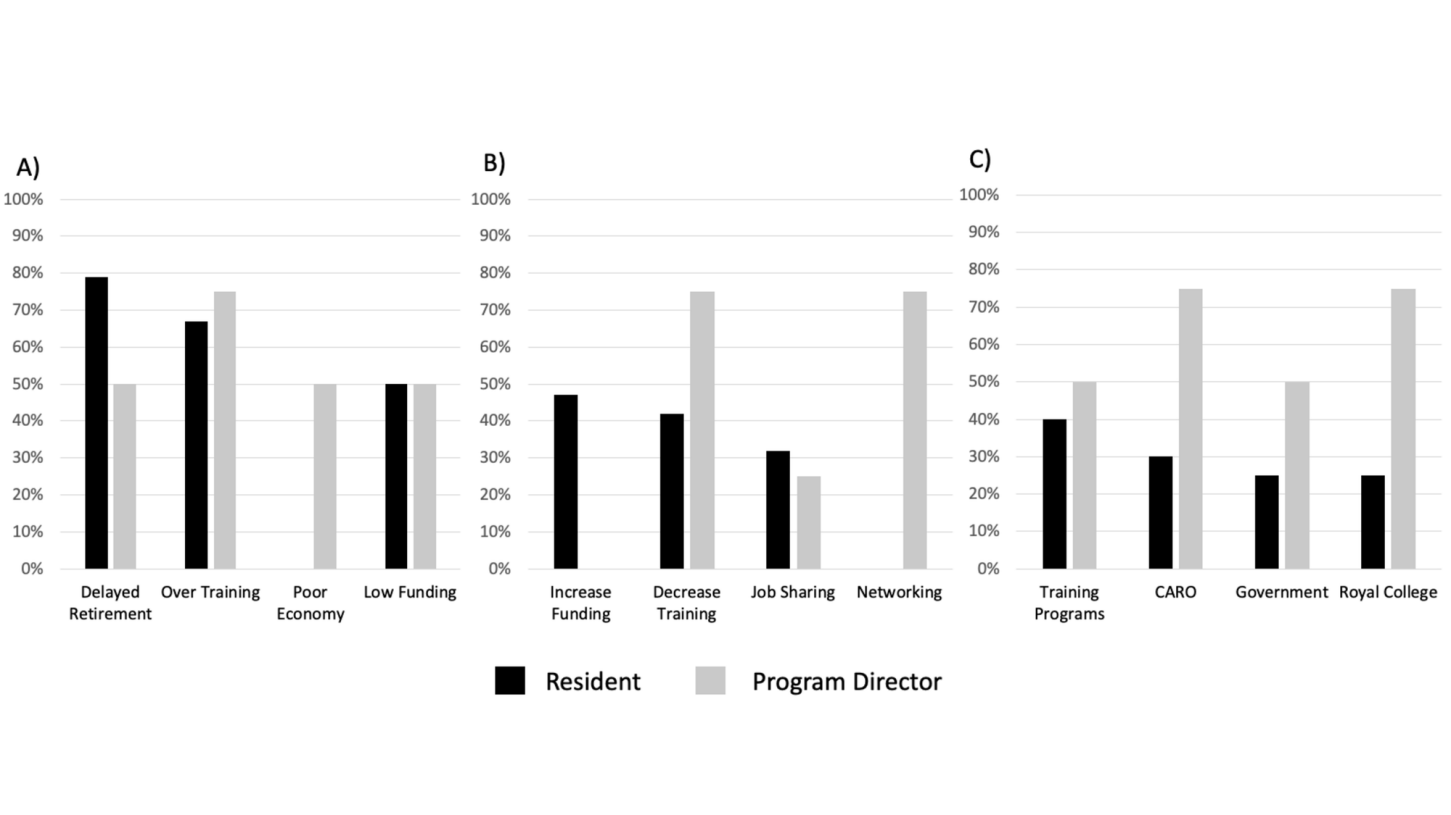
Job market perceptions of residents and program directors A) perceived drivers of job market undersupply; B) perceived solutions to address job market supply; C) perceived bodies responsible for monitoring job market supply CARO: Canadian Association of Radiation Oncology

A detailed summary of themes, subthemes, and illustrative quotes are in Table 2. Residents described the job market as poor, with a general perception that it was taking several years for graduates to find full-time employment, either doing fellowships or locums. The most commonly discussed contributors to the job market were delayed retirement (79%) and graduate oversupply (67%) (Figure 2). The most commonly cited options for improving the job market were increasing funding (47%) and decreasing the number of training positions (42%) (Figure 2). Residency training programs were the most frequently referenced body that residents felt were responsible for monitoring and regulating the job market (40%); others are listed in Figure 2.

**Table 2:** Summary of radiation oncology resident themes with example quotes

Radiation oncology job market is poor	
Delay in gaining employment after graduation	“It is virtually unheard of for residents to be offered [full time] positions…[immediately] after residency…fellowships are basically required in Canada, ultimately multi-year fellowships for no other reason than killing time until jobs become available” (participant 9)
Residents recall being discouraged from applying to radiation oncology	“At least two [recent graduates] were saying, don’t do rad onc, do med onc, or do anything else!” (participant 20)
Medical students should be made aware of job market challenges	“I do bring [the job market] up with all of them so they know. I say [radiation oncology] is a great specialty and I love everything about it…but the job situation is bad and I’m not sure it will get better. They should think about whether or not they will be ok with not having a job and working as a fellow” (participant 19)
Residents are worried about the job market	
Job market is a constant source of concern	“it’s a huge topic of conversation…it permeates everything in your life…in your work…your family and friends” (participant 3)
	“It worries me every day…I think about it all the time” (participant 19)
Job market is a stressor on personal relationships	“it's been central to discussion with my wife and…family members [weekly]” (participant 13)
Uncertainty of timing and location of future employment was the biggest stressor	“Uncertainty…that’s the big thing. Not knowing where you’re going to be in one, two, three years.” (participant 9)
	“It makes planning things like having children, buying a home…all of those things get put on hold because the job market could make [them] vanish” (participant 12)
Poor job market impacts satisfaction with training program	
Residents second-guess their decision to enter radiation oncology	“There are many days that I’m down on the job situation and…second-guess my decision to go into radiation oncology” (participant 13)
Program morale is poor	“it’s a topic of despair and frustration and discouragement. They have spent so long training and they can’t find a [job]” (participant 7)
	“Some people are feeling pretty dejected,” (participant 12)
Learning and collegial relationships are impacted	“I think overall the morale is pretty poor…everyone is so concerned about [the job market] that everyone misses out on good aspects of the residency…not focusing on the learning” (participant 17)
	“We could be a lot more collaborative…the competitiveness limits that” (participant 12)
	“People seem less willing to work together” (participant 10)

Half of the residents felt that they were well-informed about the job market prior to residency. Most of their information came from discussions with peers and preceptors. Four residents recalled being discouraged from applying to radiation oncology because of the poor job prospects. Residents who felt less informed described not fully appreciating the severity of the poor job market or having a sense of false reassurance that things would improve by the time they graduate: “everyone is…told it will…turn around by the time you graduate…talking to more senior residents, I suspect that’s being said year after year” (participant 4).

Eighty-five percent of residents discussed job market issues with medical students. Residents encouraged medical students to “prepare for uncertainty” (participant 20) and consider whether or not they are willing to accept some level of unemployment or underemployment. The three residents who did not actively discuss the job market shared a similar sentiment that unemployment and underemployment were not unique to radiation oncology, but rather a problem for multiple specialties, and therefore not a pertinent issue to highlight.

Personal Concerns

In general, residents were concerned about the current job market and how it would impact their ability to find a job. Senior residents seemed to describe a greater level of concern than junior residents. Four senior residents described spending a disproportionate amount of time dwelling on the job market. A major relationship stressor was uncertainty, particularly in relation to when and where employment might be secured after graduation. This uncertainty impacted not only the RORs’ career planning but their partners’ as well. Eight residents independently reported a concern about underemployment for their respective partners due to the uncertainty of permanent residence. Uncertainty was described by three residents as the primary reason for putting family planning on hold. Only three residents were concerned about finances and student loans, and two were concerned about finding a job in a desirable location.

Residency Training Program

Four residents reported that the lack of job prospects negatively impacted their personal satisfaction with their program. According to participant 3, “you feel a little bit abandoned” with an absence of “reward” for all of the hard work put into the training program. Seven residents contemplated switching out of the program because of the stress associated with the job market. For residents who had not contemplated switching, it was their passion for the specialty that motivated them to continue in the program, despite a difficult post-training employment scenario: “I just accepted that I really like radiation oncology and I can’t see myself doing anything different” (participant 1).

There was a general consensus that the morale among RORs in the residency training program was poor. Low morale was perceived as impacting the quality of learning. The stressors associated with the job market also impacted how residents interacted with one another. Eleven residents felt that the job market led to a competitive environment within the program that negatively impacted collaboration and relationships. Conversely, two residents felt that the job market led to increased bonding through mutual unhappiness.

There were mixed opinions as to whether or not training programs were helping residents adequately prepare for a competitive job market. Eight residents felt that their programs prepared them, 11 did not, and one was unsure. Suggestions for improving support for residents in relation to the job market included initiating career counselling earlier in training, providing support for the development of other skills that could be marketable in addition to academia, encouraging to choose electives outside of their own center, particularly in a community-based practice environment, and increasing the visibility of resident achievements across the country.

Program directors

Demographics

Four of the 13 (31%) PDs participated in the study. Three were from Western Canada. The median time spent in practice was 7.5 years (range: 5-7 years) with a median of four years working as PD (range: 1-6 years). Collectively, the four participants were responsible for 29 of the 113 RORs (26%) across the country.

Perceptions of the Job Market

A summary of themes, subthemes, and quotes are described in Table 3. All four PDs described a shortage of jobs. Each PD described a pattern of graduates completing multiple fellowships before finding full-time employment. Two described this pattern as a national problem and three felt that it made graduates over-qualified.

**Table 3:** Summary of radiation oncology program director themes with example quotes

Sense of responsibility to trainees	
Profession is obligated to support trainees in their transition into the workforce	“We still need to take care of everyone in our profession. There is a pervasive attitude…that the cream will rise to the top and the rest will fall where they may. I don’t agree with that. If…students are accepted into a program, then there should be…efforts to ensure that jobs are available, based on public need…anything else is irresponsible” (participant 4)
Feel like a parental figure	“I feel responsible for my residents. Like their [parental figure]. When they graduate and can’t get jobs, I feel like I should be doing something about it” (participant 1)
Job market impacts job satisfaction	
Source of stress	“I find it a real shame…I feel stress for them…it is kind of depressing” (participant 3)
Unequipped to provide required support	“We…do a fairly good job with mentoring, but…I’m lacking in the ability to counsel people about how to find a job” (participant 2)
	“I have no career counselling training” (participant 4)
Job market is impacting resident well-being	
Residents are worried	“The residents are worried and scared…there is only uncertainty…incredibly stressful for all of them” (participant 1)
	“Many of them are quite emotional about it…they have multiple commitments in their lives that make it…complicated” (participant 3)
Residents second guess career choice	“I think that they are always second-guessing their choice of career... I think they feel very frustrated and vulnerable” (participant 2)
Morale is poor	“Morale has been identified as a major concern among our residents. [It] is directly related to the job market…I think it is contagious and…difficult for even the most enthusiastic, optimistic resident to maintain morale” (anonymous)
Residents have been brought together by common stressor	“The residents bring [each other] up because it is a shared experience…we have a very tight-knit group…they…support one another” (participant 1)
	“I see…admirable things…I think they try very hard not to step on each other’s toes” (participant 3)

Three PDs believed that delayed employment was directly due to over-training (Figure 2). Three PDs felt that national organizations such as CARO and RCPSC should be responsible for monitoring the job market and recommend adjustments in trainee numbers (Figure 2). Multiple solutions for correcting the job market were proposed. Three PDs felt that trainee numbers could be aligned with workforce demand projections using a “needs-based prediction model.” Three supported resident networking outside of their training program. Two felt that internationally sponsored trainees should be limited. One felt strongly that retirement or job sharing should be encouraged, whereas another described a need to study and respond to retirement patterns.

Impact on Role as Program Director

Each PD was definitive in reporting that the job market negatively impacted their personal satisfaction with their position. There was a sense of professional responsibility to support their graduates in finding a job. Job market difficulties were a source of stress, with two PDs describing feeling like a parental figure. Each PD also described a responsibility to discuss the job market with medical students: “I’m as honest as I can be…I’m absolutely upfront about it” (participant 2); “yes I have to…[otherwise] it is unethical” (participant 4). In the experience of two PDs, the medical students often initiated the conversation and seemed to already be informed about job shortages. One PD felt that this knowledge had led to a decrease in the number and quality of applicants. Each PD described feeling unequipped or underpowered to support trainees in preparing for entering the workforce amidst the job shortages.

Perceived Impact on Residents

When asked how residents feel about the job market, PDs were unanimous in describing “stress,” “fear,” and “concern.” Resident morale is described as “poor” by all PDs. Two PDs described the morale being worse for senior residents compared to junior residents. Despite the low morale and competition for jobs, PDs did not feel that there was a competitive environment in their programs, but rather a supportive one unified by a common stressor.

## Discussion

To our knowledge, this is the first analysis of ROR perception of the job market and its impact on well-being, and the first to include PDs. This assessment in 2014/2015 identified that the majority of the 20 Canadian trainees interviewed were concerned about the job market (60%), and described it as impacting their personal (60%) and professional (55%) relationships. PDs perceived the job market to be negatively impacting resident well-being and their own job satisfaction. Unique to this study was the attempt to describe the “lived experience” of residents as they progressed through a rigorous training program facing the prospect of unemployment upon graduation. These findings have important implications, not only for Canadian radiation oncology training programs but for radiation oncology programs worldwide and other medical specialties experiencing fluctuation in job availability.

The radiation oncology job market fluctuation has been documented internationally. In 2010, a US workforce forecast concluded that the demand for radiation oncologists would exceed supply and supported an increase in training numbers [11]. Years later, concerns of underemployment of recent graduates were raised [12]. In 2015, 61% of US graduates felt that the job market was worse than what they had originally anticipated, and one-third were unable to obtain employment in their geographic region of preference [13]. The potential impact of these job shortages on well-being and morale was not explored. Likewise, in Australia, trainee oversupply was described as a critical issue [14]. Similar to our Canadian trainees, a 2014 survey of Australian residents described job prospects as their greatest source of stress with 94% concerned about job availability [15].

Employment challenges are also present in multiple Canadian medical specialties. There are several similarities identified between RORs and trainees from other sub-specialties with regard to their perceptions of the job market. For example, one-quarter of Canadian general surgery graduates were “dissatisfied or neutral” about their employment situation [16]. Example quotes from orthopedic and cardiothoracic residents, respectively, resonate with ROR comments about their disappointment regarding job prospects: “extremely discouraging to have gone through 15 years of post-secondary education and not to have full-time employment,”; “it is very disheartening to train for the amount of time that I have trained and have no guarantee for a job.” [17,18]. Interestingly, concerns about educational debt aggravated by employment and financial uncertainties were more prominent amongst surgical residents compared to our cohort [17-19]. Some of the consequences of the difficult job market described by RORs were similarly described by surgical residents. For example, a pattern of undertaking fellowships to improve employability has been reported by Canadian ophthalmology and orthopedic trainees [17,20]. Also, there was a decline in cardiothoracic trainees recommending the training program to prospective medical students; additionally, over one-quarter of Canadian surgical trainees considered leaving their specialty, partly due to concerns regarding unemployment or underemployment [19,21].

The literature also describes the impact of turbulent job markets on the professional and personal well-being of university students and graduates in the general population. Poor perceptions of employability, for example, have a negative impact on physical health, job performance, and life satisfaction [22]. This body of literature is consistent with our findings that the job market contributed to poor morale, decreased satisfaction with the training program, and disruption of personal and professional relationships. These findings are important to consider in the context of physician burnout. Oncologists, in particular, are at an increased risk for burnout due to compassion fatigue, stressful patient interactions, and increasing workload; and residents, in general, are at a higher risk of burnout than medical students, early-career physicians, and the general population [23,24]. Although our study did not formally assess burnout, a prior survey of Canadian oncology residents in 2017 identified a burnout rate of 42%, and the most commonly reported stressor was future career planning (79%) [25].

The results of this study should be considered in the context of its strengths and limitations. Our response rate is much lower than prior Canadian ROR surveys (18% vs 52%) and does not represent residents from Quebec or Atlantic Canada. This response rate could be explained by the logistical effort and time commitment required to participate in the interview and the lack of anonymity in discussing potentially sensitive topics. While our sample size could be considered small in comparison to prior surveys, our study on residents reached “saturation”, the point at which new ideas or themes are no longer identified, after 17 interviews. Saturation for PDs was not achieved. The relatively small sample size could also lead to questions about the generalizability of our findings to RORs and PDs across Canada; however, for the purposes of this qualitative study, a smaller sample size allowed for a more in-depth evaluation of each individual case in an effort to explore the “lived experience” of each participant [26]. While the one-on-one interviews by multiple investigators aimed to understand the participants’ experience from their point of view and decrease the likelihood of researcher bias, there was no prior training of interviewers or interim audit of transcripts to ensure consistency in interviewing technique [26]. The open-ended questions and semi-structured format, however, aimed to provide the opportunity for a full expression of ideas and feelings in a less restricted manner than a rigid multiple-choice survey. One should also consider the possibility of participation bias in our study; however, the similar themes independently identified by residents and PDs enhance the robustness of our findings and are consistent with themes previously identified over time in the literature in Canada [1-3,5]. Member checking, whereby RORs and PDs have the opportunity to review our perceptions for accuracy and further clarification, could have further strengthened our conclusions but was not performed in this study.

Since the completion of this study, the Canadian radiation oncology job market has modestly improved, but this does not diminish the importance or relevance of our findings [27]. Employment difficulties continue to be reported and appear to be cyclical as highlighted by the waxing and waning job market over the past four decades. Unfortunately, the 2019 RCPSC Employment Study reported that the proportion of medical specialists who are unemployed at the time of certification has increased to 19% [28]. Radiation oncology graduates continue to be over-represented in this cohort. The possible down-stream and latent effects of these job market fluctuations must also be considered. For example, the perceived job market shortages can impact recruitment of medical students to the specialty as evident in 1997 and again in 2017 with only nine applicants across Canada ranking radiation oncology as their first choice (a 67% reduction compared to 2008) [3,29]. Similarly, in the US, medical student interest in radiation oncology has been declining since 2014, and some members believe this is because students perceive the job market to be unfavorable [30]. Additionally, as radiation oncology is not alone in its manpower struggles, the findings of this study may provide insight into the challenges that other specialist trainees are experiencing and form a framework for further research in this area.

## Conclusions

Radiation oncology residents who experience significant uncertainty with regard to future job prospects report concerns about finding employment, which impacts their personal and professional well-being. While fluctuating market forces continue to impact the job market for our specialty, regulatory bodies, undergraduate and post-graduate medical educators would be wise to collaborate on discussing possible strategies for this cyclical issue. In the interim, training programs could take the opportunity to incorporate career counselling, mentorship programs, and resiliency training into their curriculum to address resident morale and wellness. Failure to do so may jeopardize our ability to recruit and retain skilled individuals, thereby threatening the sustainability, quality, and wellness of our workforce.
